# Comparative Plastome Analysis of Two *Viburnum* Species from East Asian Islands: Genome Conservation, Localized Divergence, and Plastome-Based Phylogenetic Placement

**DOI:** 10.3390/ijms27219467

**Published:** 2026-10-23

**Authors:** Dongyue Jiang, Xin Shen

**Affiliations:** Institute of Tree Breeding, Zhejiang Academy of Forestry, 399 Liuhe Road, Hangzhou 310023, China; jdyzjforestry@163.com

**Keywords:** *Viburnum*, chloroplast genome, comparative genomics, phylogenetic analysis

## Abstract

The genus *Viburnum* represents an evolutionarily informative lineage of woody angiosperms, but plastome variation in the two focal species remains insufficiently characterized. In this study, we assembled and compared one complete plastome from each of *V. suspensum* and *V. taitoense* to characterize genome conservation, sequence divergence, codon-usage patterns, and plastome-based phylogenetic placement. The complete plastomes of *V. suspensum* and *V. taitoense* were 158,577 bp and 158,544 bp in length, respectively, and both contained 133 annotated genes with an identical quadripartite architecture and the same GC content (38.1%). Comparative analysis with 52 additional publicly available *Viburnum* plastome records revealed highly conserved genome organization across the sampled records, whereas sequence divergence was unevenly distributed among genomic regions. The inverted-repeat regions showed higher conservation than the single-copy regions, and sliding-window analysis of 98 plastomes identified the *ndhF–rpl32* intergenic region as the only major variable region exceeding the predefined threshold (maximum Pi = 0.06171). Both focal plastomes exhibited highly similar codon-usage patterns, with 29 preferred codons, 28 ending in A or U and UUG as the only preferred codon ending in G, and weak overall codon bias (ENC = 50.26 and 50.24). Complete-plastome phylogenetic analysis recovered the sampled plastomes of *V. suspensum* and *V. taitoense* as a sister pair, although this node received only moderate bootstrap support (BS = 72). These findings provide plastome-level evidence for the sampled accessions of *V. suspensum* and *V. taitoense* and establish a basis for broader population-level and phylogenomic investigations.

## 1. Introduction

*Viburnum* L. (Viburnaceae) is a diverse lineage of woody angiosperms comprising approximately 160 recognized extant species of evergreen or deciduous shrubs and small trees distributed mainly across temperate and subtropical regions of the Northern Hemisphere. Eastern Asia is the principal center of species and phylogenetic diversity in the genus, with approximately 89 species recognized in the region under a recent global biogeographic treatment [1]. China constitutes a major component of this diversity center, harboring 73 species, including 45 endemic species [2]. Across East Asia, *Viburnum* taxa occur from continental China and the Korean Peninsula through the Japanese archipelago and the Ryukyu Islands to Taiwan, mainly in temperate and subtropical forest habitats [1,2,3,4]. The Americas constitute another major center of diversity [3,4]. Many *Viburnum* species possess considerable ornamental, ecological, and medicinal value, and several taxa are widely cultivated in urban landscapes and gardens worldwide or utilized in traditional medicine because of their diverse bioactive compounds [5]. Beyond their practical importance, the broad geographic distribution, repeated occupation of contrasting forest biomes, and extensive variation in reproductive and vegetative traits have made *Viburnum* an informative system for studying plant diversification, biogeography, and trait evolution [6]. Molecular studies based on plastid, nuclear, and phylogenomic datasets have established the principal evolutionary framework of the genus and revealed major lineage diversification patterns [7,8]. However, relationships among some rapidly diversified groups and deeper evolutionary relationships within the genus remain unresolved, highlighting the need for additional genomic evidence and broader comparative sampling.

Complete chloroplast genomes provide a useful genomic scale for addressing such questions because they combine a conserved plastome architecture with localized sequence divergence. Plastomes of many photosynthetic angiosperms retain a quadripartite organization and relatively stable gene content and gene order, but this conserved plastome backbone is not universal and does not imply sequence or structural stasis [9,10,11]. Substitutions, indels, inverted-repeat boundary changes, and other local features are distributed heterogeneously and can preserve evolutionary information even among plastomes with similar overall organization. This combination of conserved architecture and heterogeneous sequence evolution makes plastomes suitable for investigating evolutionary patterns among recently diversified lineages. Accordingly, plastome-scale datasets have increased phylogenetic information relative to sparse plastid markers in many plant groups [12,13,14]. However, plastome topologies describe plastid evolutionary history and may differ from relationships inferred from nuclear data; even strongly resolved plastid trees should therefore not be treated automatically as definitive species trees [15].

Comparative studies of *Viburnum* plastomes have begun to establish the genomic context for addressing these issues. Across sampled taxa, complete plastomes generally retain the typical quadripartite structure and broadly conserved genome organization while exhibiting localized sequence divergence in inverted-repeat boundaries, noncoding regions, and other variable portions of the genome [16,17,18,19]. Complete-plastome datasets have also increased the evolutionary information available for evaluating relationships among major *Viburnum* groups, although some placements remain sensitive to taxon sampling, classification schemes, or the genomic data analyzed [8,16,20]. Comparative evaluation of coding-region composition provides a complementary perspective on plastome conservation, but codon-usage patterns cannot be attributed to a single evolutionary force based on one diagnostic alone. Despite recent progress, the plastomes of *V. suspensum* and *V. taitoense* have not been directly compared within a unified analytical framework. These species were selected because they occur in two geographically separated East Asian island systems—the Ryukyu Islands and Taiwan, respectively—and because their plastome characteristics and phylogenetic placements remain insufficiently compared. The two focal specimens were taxonomically verified to ensure reliable sample identity. Although geographic separation may contribute to lineage differentiation over evolutionary time, the present sampling was not designed to test its effects on gene flow or to establish a causal relationship between island isolation and the observed plastome patterns. Instead, we characterize one sampled plastome from each species and integrate analyses of genome structure, localized sequence divergence, codon-usage patterns, and plastome-based phylogenetic placement. Comparisons with broader *Viburnum* datasets provide a genus-level context for interpreting the plastome characteristics of the two focal samples.

This study had three objectives. First, we assembled and characterized the complete plastomes of *V. suspensum* and *V. taitoense* and compared their genome organization and sequence conservation using Dataset I, which comprised 54 *Viburnum* plastome records. Second, we characterized codon-usage patterns in the two focal plastomes and evaluated nucleotide diversity using the 98-record Dataset II, treating localized sequence variation as candidate evolutionary information rather than as evidence of accelerated or adaptive evolution. Third, we inferred the plastome-based phylogenetic placement of the focal samples using Dataset III, which comprised 101 plastome records, including 98 *Viburnum* records and three *Sambucus* outgroups. Complete plastomes can provide more informative characters than individual plastid loci while still representing a single plastid history [21,22]. Together, these analyses evaluate plastome conservation, localized sequence divergence, codon-usage patterns, and plastome-based phylogenetic placement within the explicitly defined sampling framework.

## 2. Results

### 2.1. Chloroplast Genome Features of V. suspensum and V. taitoense

**Figure 1:**
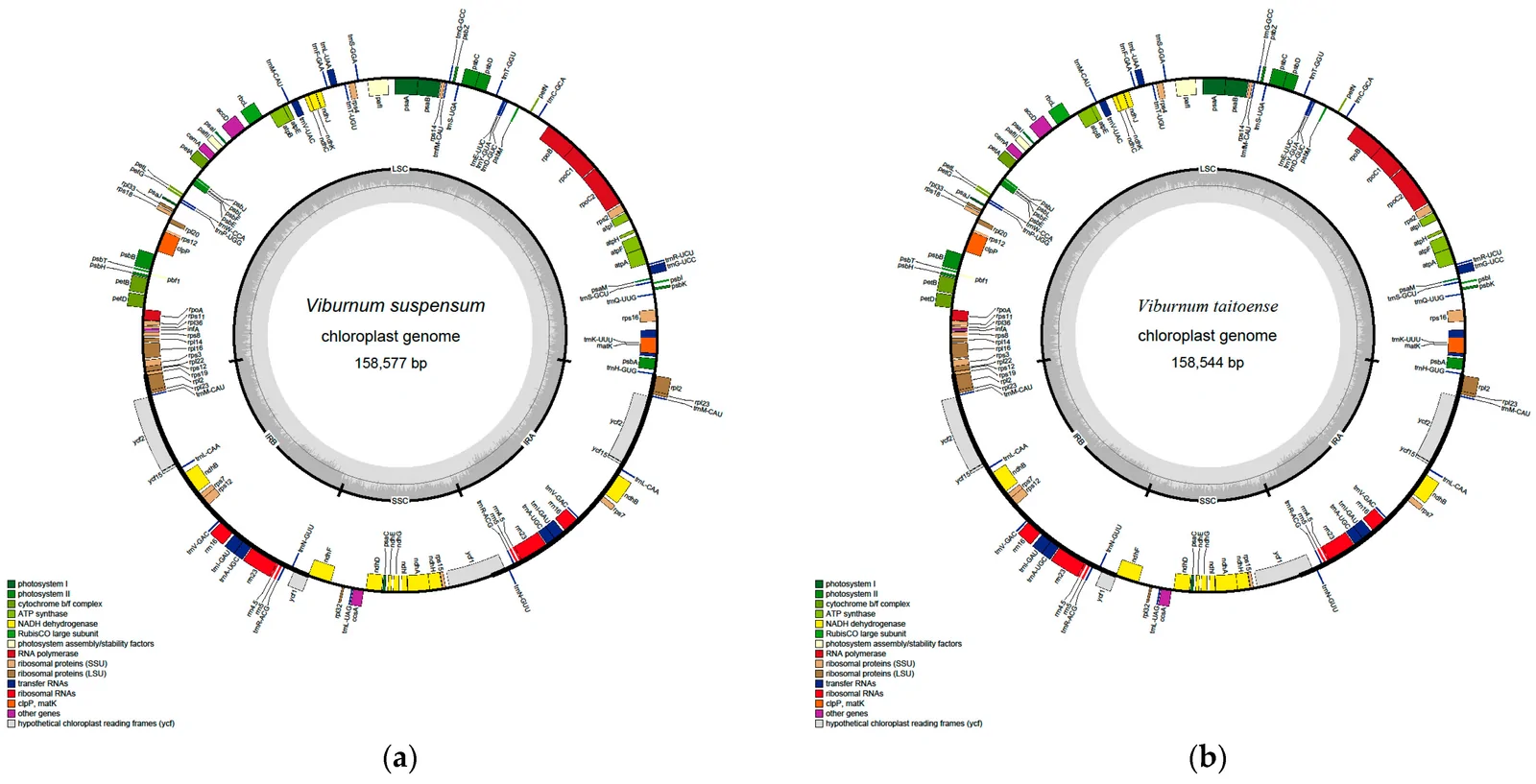
Gene maps of the chloroplast genomes of *Viburnum suspensum* and *V. taitoense*. (**a**) *V. suspensum*; (**b**) *V. taitoense*. Genes located outside the circle are transcribed clockwise, whereas those inside the circle are transcribed counterclockwise. The dark and light gray bars in the inner circle indicate the GC and AT contents of the chloroplast genome, respectively.

The complete chloroplast genomes of *V. suspensum* and *V. taitoense* were successfully assembled using high-quality paired-end sequencing reads. For *V. suspensum*, a total of 63,826,390 clean paired-end reads were obtained, of which 1,537,310 reads (2.41%) were identified as chloroplast-derived reads, resulting in an average sequencing depth of approximately 1454×. For *V. taitoense*, 67,824,188 clean paired-end reads were generated, including 994,029 chloroplast-derived reads (1.47%), with an average sequencing depth of approximately 940×. Both chloroplast genomes exhibited the typical quadripartite structure of angiosperm plastomes (Figure 1), consisting of one large single-copy (LSC) region, one small single-copy (SSC) region, and two inverted repeat (IR) regions.

The chloroplast genome of *V. suspensum* was 158,577 bp in length, comprising an 87,086 bp LSC region, an 18,423 bp SSC region, and two 26,534 bp IR regions. Similarly, the chloroplast genome of *V. taitoense* was 158,544 bp in length, consisting of an 87,036 bp LSC region, an 18,422 bp SSC region, and two 26,543 bp IR regions. The two plastomes showed highly similar genomic architectures, differing by only 33 bp in total genome length. The overall GC content was 38.1% in both species, with identical GC contents in the LSC (36.4%), SSC (32.1%), and IR (43.0%) regions.

Both chloroplast genomes contained 133 annotated genes, including 88 protein-coding genes, 37 tRNA genes, and eight rRNA genes, representing 114 unique gene species (Table 1). The gene composition was identical between the two species. Among these, 17 gene species were duplicated in the IR regions, including seven protein-coding genes, six tRNA genes, and four rRNA genes. In addition, *trnM-CAU* was present in three copies in both plastomes, with one copy located in the LSC region and one copy in each of the two IR regions. Excluding the trans-spliced *rps12*, 15 genes contained a single intron, whereas *pafI* and *clpP* each contained two introns. The *rps12* gene comprised one 5′ exon in the LSC region and a duplicated 3′ exon region in the two IRs, with each 3′ region containing exons 2 and 3 (Figure S1).

### 2.2. Comparative Analysis of Chloroplast Genome Structure and Sequence Divergence

Dataset I comprised 54 *Viburnum* plastome records representing 54 complete taxon labels: 52 publicly available records and the two plastomes newly sequenced in this study (Table S1). The mVISTA comparison showed a highly conserved genome organization across the sampled taxa, with no obvious large-scale rearrangements. The inverted-repeat (IR) regions were more conserved than the large single-copy (LSC) and small single-copy (SSC) regions, whereas noncoding regions showed greater sequence divergence than coding regions (Figure S2).

Across Dataset I, plastome lengths ranged from 157,433 to 159,015 bp; the LSC, SSC, IRb, and IRa regions ranged from 86,426 to 87,491 bp, 18,199 to 19,170 bp, 26,124 to 26,558 bp, and 26,124 to 26,558 bp, respectively (Figure S3; Table S2). At JLB, the *rps19* feature crossed the LSC/IRb junction in all 54 plastome records. In 50 records, 32–34 bp of *rps19* extended into IRb. The exceptions were *V. dentatum* (36 bp), *V. cylindricum* (49 bp), and the two newly sequenced plastomes of *V. suspensum* and *V. taitoense* (63 bp each). The corresponding portions within the LSC ranged from 216 to 247 bp (Figure S3; Table S2). At JSB, a *ycf1* feature crossed the junction in 39 records, was adjacent to the junction in two records, and was not annotated near the junction in 13 records. The two adjacent features were located within IRb in *V. luzonicum* (MT507589.1), 17 bp from JSB, and *V. setigerum* (MT507585.1), 6 bp from JSB. Among the crossing records, the portion within IRb ranged from 1311 bp in *V. tinus* (OR722540.1) to 1466 bp in both *V. keteleeri* (C_AA478700.1) and *V. keteleeri* ‘Sterile’ (C_AA478699.1), whereas the portion within SSC ranged from 4 bp in *V. melanocarpum* (MT507587.1) to 75 bp in *V. tinus* (OR722540.1). Because the annotation status of the JSB-associated sequences was not consistent among records, they are conservatively referred to as ‘*ycf1* features’ rather than uniformly classified as complete genes or pseudogenes. The *ndhF* annotation crossed JSB in four records, with 8–65 bp in IRb and 2212–2230 bp in the SSC; it was adjacent to JSB on the SSC side in 45 records, at distances of 0–122 bp, and was not annotated near JSB in five records. At JSA, a *ycf1* feature crossed the junction in 53 records, with 4183–4644 bp in the SSC and 1025–1466 bp in IRa; no *ycf1* feature was annotated near JSA in one record. At JLA, the *rpl2* feature was located entirely within IRa and adjacent to the junction in all 54 records, at distances ranging from 90 to 121 bp. The minimum distance of 90 bp was observed in *V. betulifolium*, *V. awabuki*, *V. cinnamomifolium*, and *V. melanocarpum*. A distance of 107 bp was recorded in *V. cylindricum*, whereas the maximum distance of 121 bp occurred in the two newly sequenced plastomes of *V. taitoense* and *V. suspensum*. The *trnH-GUG* annotation crossed JLA in one record, with 1 bp in IRa and 74 bp in the LSC; it was adjacent to JLA on the LSC side in 51 records, at distances of 0–375 bp, and was not annotated near JLA in two records.

### 2.3. Analysis of Repetitive Sequences and SSRs

Repeat analysis of the complete plastomes identified 45 and 44 short repeat pairs in *V. suspensum* and *V. taitoense*, respectively, after excluding the canonical long IRa–IRb repeat pair (Table 2). These repeats consisted of 19 direct and 26 palindromic repeat pairs in *V. suspensum* and 18 direct and 26 palindromic repeat pairs in *V. taitoense*. Repeat lengths ranged from 30 to 64 bp, indicating that short repetitive elements predominated in both plastomes.

The genomic distributions of the repeat pairs were also similar between the two plastomes. In *V. suspensum*, 22 repeat pairs were associated with the IR regions, 12 with the LSC region, one with the SSC region, and 10 were classified as cross-region. The corresponding numbers in *V. taitoense* were 22, 11, one, and 10, respectively.

At the occurrence level, the 45 and 44 repeat pairs corresponded to 90 and 88 repeat occurrences, respectively. Both plastomes contained 38 repeat occurrences within CDS regions, 20 within introns, four crossing feature boundaries, and two within tRNA genes. Intergenic regions contained 26 repeat occurrences in *V. suspensum* and 24 in *V. taitoense*, and no repeat occurrences were located within rRNA genes (Table 2; Table S3). Overall, the two plastomes exhibited closely comparable repeat-type, regional, and functional distributions.

Simple sequence repeats (SSRs) were further characterized to evaluate the repetitive sequence features of the two *Viburnum* chloroplast genomes. A total of 42 and 41 SSR loci were identified in the chloroplast genomes of *V. suspensum* and *V. taitoense*, respectively (Table 3). All detected SSRs belonged to mononucleotide repeats, with no di-, tri-, tetra-, penta-, or hexanucleotide repeats identified. The SSR composition was strongly biased toward A/T-rich motifs, with 41 A/T-type and one C/G-type SSR detected in *V. suspensum*, and 40 A/T-type and one C/G-type SSR detected in *V. taitoense*, indicating a highly conserved AT-rich SSR composition between the two insular *Viburnum* lineages. The genomic distribution patterns of SSRs were also highly similar between the two plastomes. In *V. suspensum*, SSRs were mainly located in the LSC region (30 loci), followed by the SSC (six loci) and IR regions (six loci). Similarly, *V. taitoense* contained 27, eight, and six SSR loci in the LSC, SSC, and IR regions, respectively (Table S4). Regarding functional location, 11 and 12 SSRs occurred within CDS regions in *V. suspensum* and *V. taitoense*, respectively; four and five occurred within introns; zero and one occurred within tRNA genes; and 27 and 23 occurred within intergenic regions. No SSRs were located within rRNA genes or across feature boundaries in either plastome (Table 3; Table S4).

### 2.4. Codon Usage Bias

Codon usage patterns were analyzed based on the protein-coding genes of the two newly sequenced *Viburnum* chloroplast genomes. A total of 26,971 and 26,965 codons, including stop codons, were identified from the coding sequences of *V. suspensum* and *V. taitoense*, respectively. The overall codon usage patterns were highly similar between the two plastomes, with nearly identical values observed for major codon usage parameters. The GC contents of both chloroplast genomes were 38.1%, while the GC contents at synonymous third codon positions (GC3s), as calculated by CodonW, were 27.7%, indicating a preference for A/T-rich synonymous codons.

**Figure 2:**
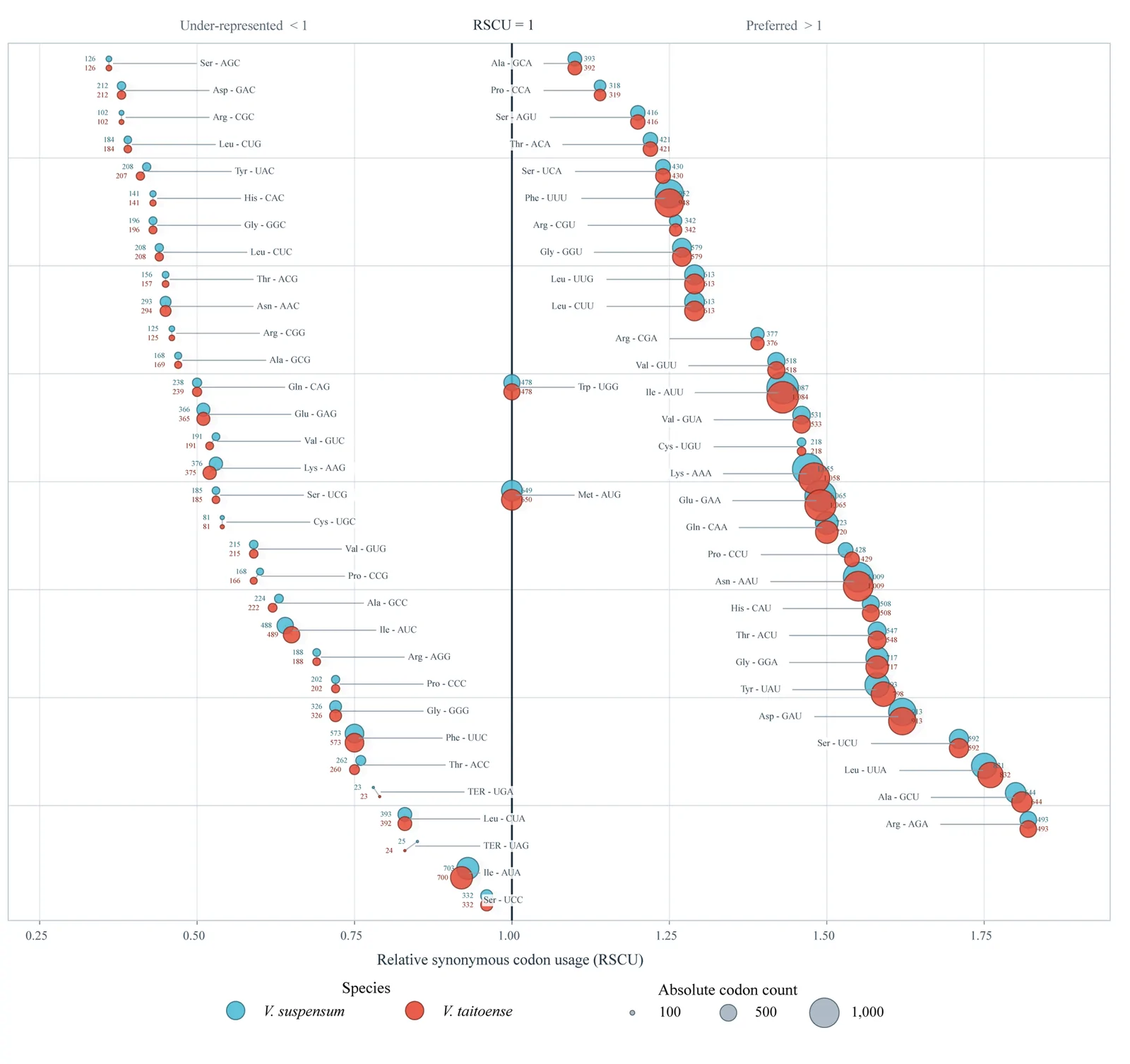
Relative synonymous codon usage (RSCU) in the chloroplast genomes of *V. suspensum* and *V. taitoense*.

Relative synonymous codon usage (RSCU) analysis further revealed highly similar codon preferences between the two focal plastomes (Figure 2; Table S5). After excluding stop codons from the identification and enumeration of preferred codons, 29 codons had RSCU values greater than 1 in both *V. suspensum* and *V. taitoense*. Of these, 28 ended in A or U, whereas UUG was the only preferred codon ending in G. The effective number of codons was 50.26 for *V. suspensum* and 50.24 for *V. taitoense*, indicating a relatively weak codon usage bias in both chloroplast genomes. Together, the highly similar RSCU profiles, GC composition, and ENC values suggest that the two focal *Viburnum* plastomes share similar codon-usage patterns.

**Figure 3:**
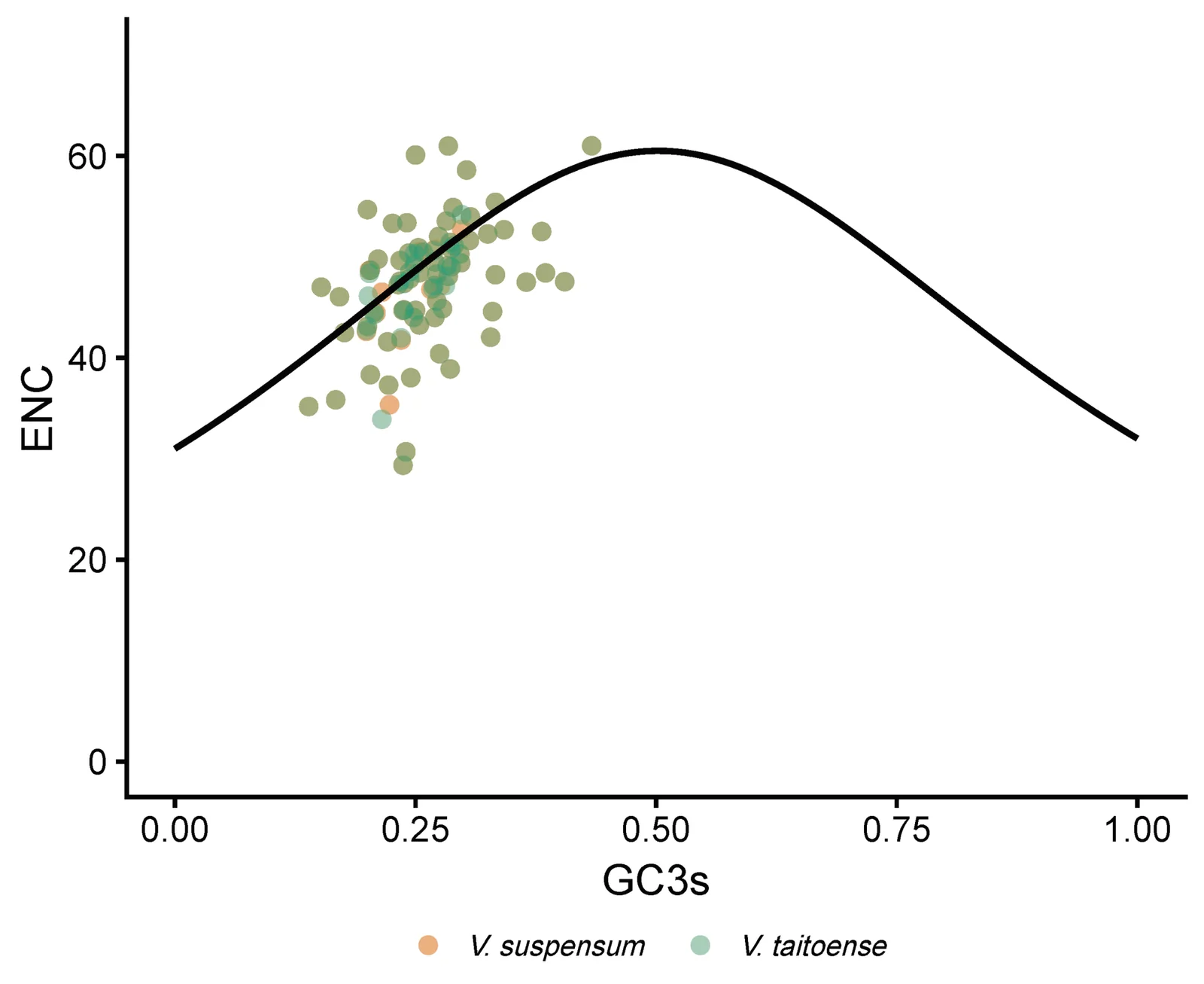
ENC–GC3s plot analysis of codon usage bias in the chloroplast genomes of *V. suspensum* and *V. taitoense*. The ENC–GC3s plot includes 80 genes with valid ENC estimates for each plastome; *psbT* was excluded because no valid ENC value was obtained.

To further evaluate the factors influencing codon usage patterns, ENC–GC3s analysis was performed using the 80 genes with valid ENC estimates from each plastome (Figure 3). The ENC values ranged from 29.36 to 61.00 in both species, and GC3s values ranged from 0.139 to 0.433. Most genes were distributed within the ranges of GC3s values of approximately 0.20–0.35 and ENC values of 40–55. Of the 80 genes with valid ENC estimates in each plastome, 51 genes (63.8%) were located below the expected ENC curve, whereas 29 genes (36.3%) were located above it.

The ENC values varied among different protein-coding genes, and the two species showed identical distributions of genes with relatively high and low ENC values. Genes including *psbJ*, *petN*, *rpl36*, *rps8*, and *rpl16* exhibited lower ENC values, indicating stronger codon usage preferences, whereas *ycf15*, *infA*, and *psaC* showed higher ENC values, reflecting weaker codon preferences. These results revealed variation in codon usage patterns among different functional genes in both *Viburnum* chloroplast genomes.

**Figure 4:**
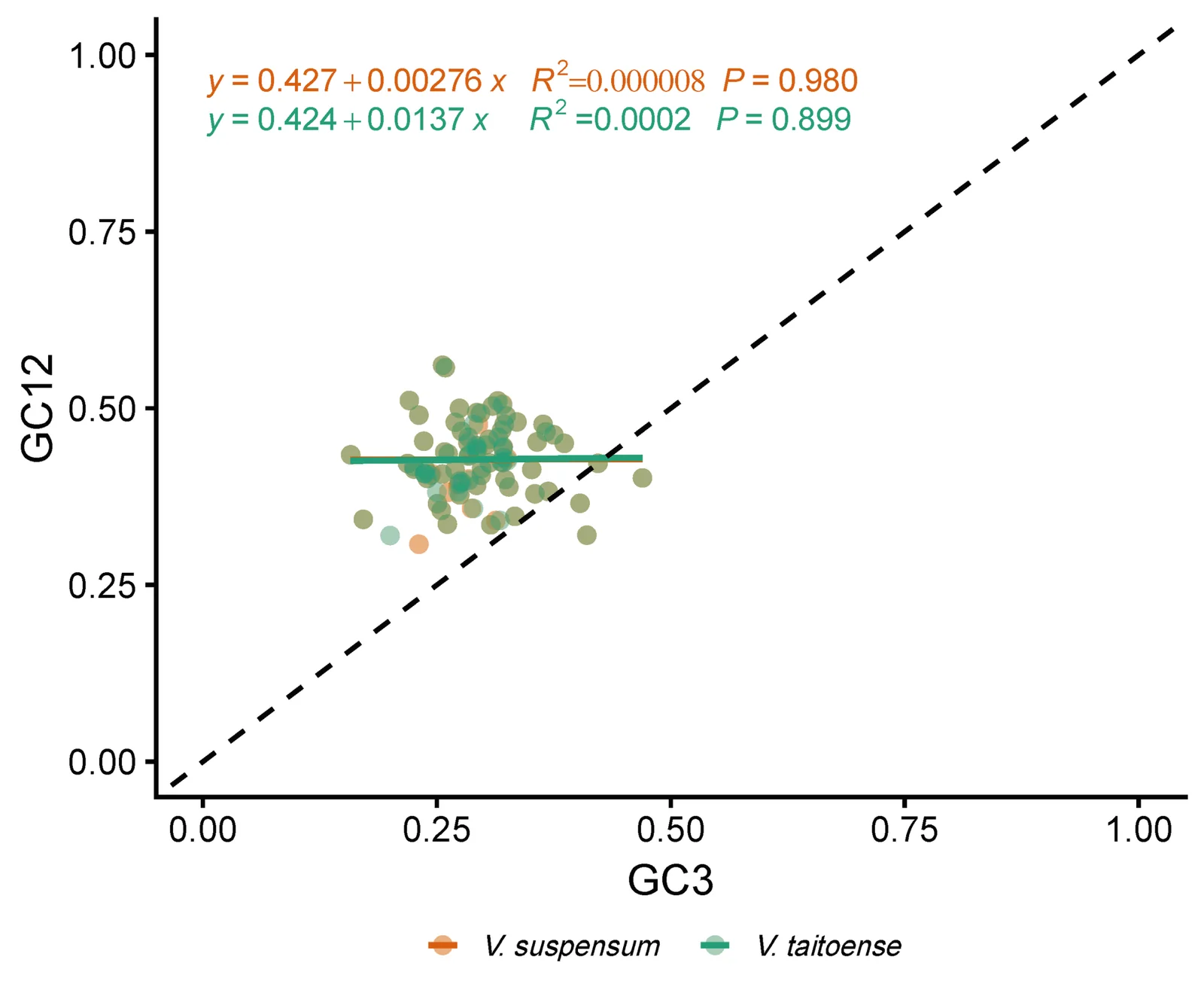
Neutrality plot analysis of codon usage bias in the chloroplast genomes of *V. suspensum* and *V. taitoense*.

Neutrality analysis was further performed to evaluate the contribution of mutation pressure to codon usage variation (Figure 4). No significant correlation was detected between GC3 and GC12 in either species. In *V. suspensum*, the regression slope between GC3 and GC12 was 0.0028, with an R^2^ value of 8.1 × 10^−6^ (*P* = 0.9799), whereas *V. taitoense* showed a slope of 0.0137, an R^2^ value of 2.1 × 10^−4^ (*P* = 0.8987). The slopes in both species were close to zero, indicating that variation in the third codon position had limited effects on the overall nucleotide composition of protein-coding genes.

**Figure 5:**
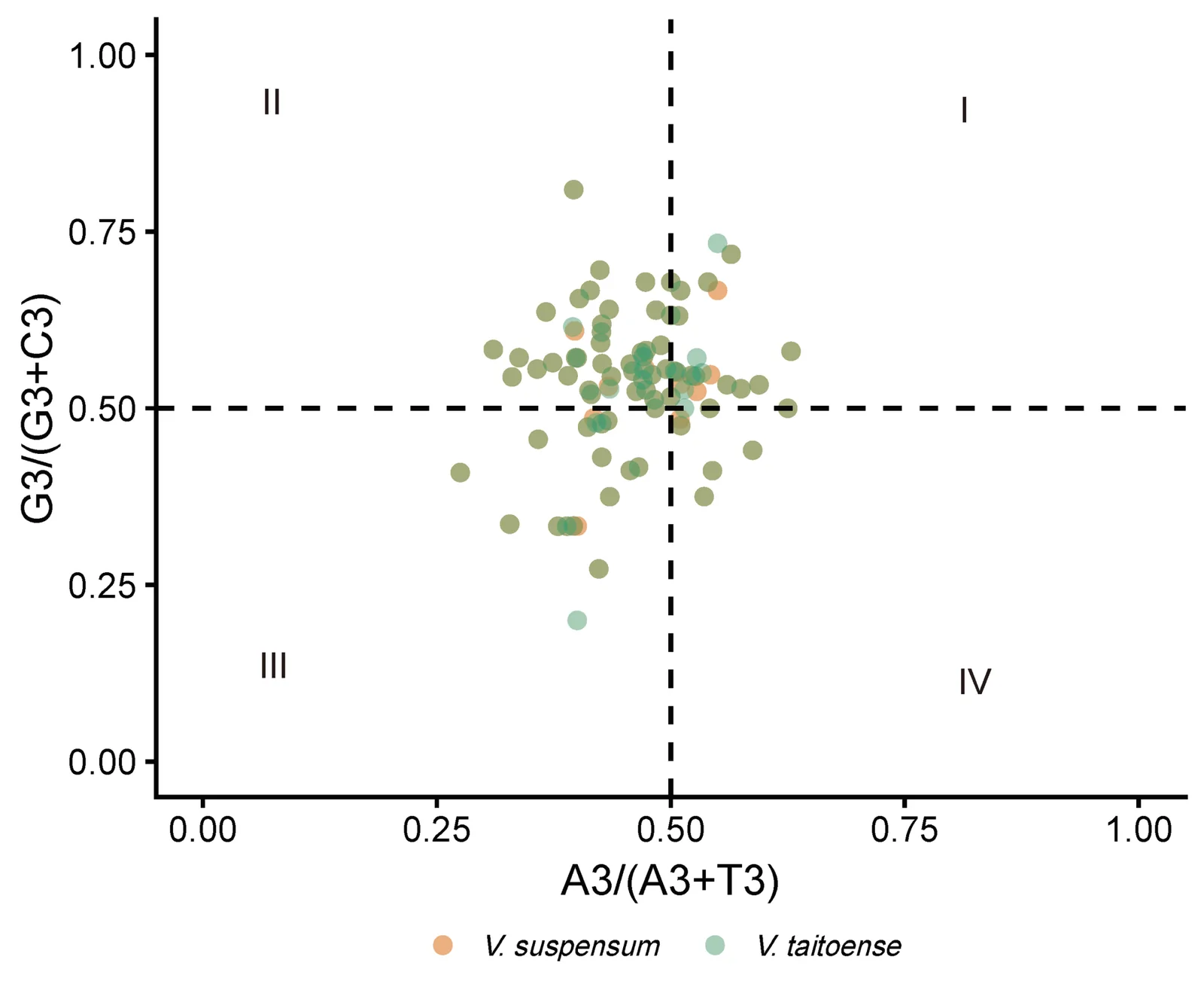
PR2-bias plot analysis of codon usage bias in the chloroplast genomes of *V. suspensum* and *V. taitoense*.

PR2 analysis was conducted to examine nucleotide bias at the third codon position (Figure 5). The distribution patterns of the two species were highly similar, and most genes deviated from the theoretical equilibrium point (0.5, 0.5), indicating an unequal usage of A/T and G/C bases at the third codon position. The mean values of A3/(A3 + T3) and G3/(G3 + C3) were 0.4589 and 0.5365 in *V. suspensum*, and 0.4589 and 0.5363 in *V. taitoense*, respectively. In both species, A3/(A3 + T3) values were lower than 0.5, whereas G3/(G3 + C3) values were higher than 0.5, indicating a preference for T and G at the third codon position. Furthermore, identical quadrant distributions were observed between the two species, with 38 genes located in quadrant II, 16 genes in quadrant I, 16 genes in quadrant III, and 11 genes in quadrant IV.

### 2.5. Sequence Divergence and the Major Variable Region

**Figure 6:**
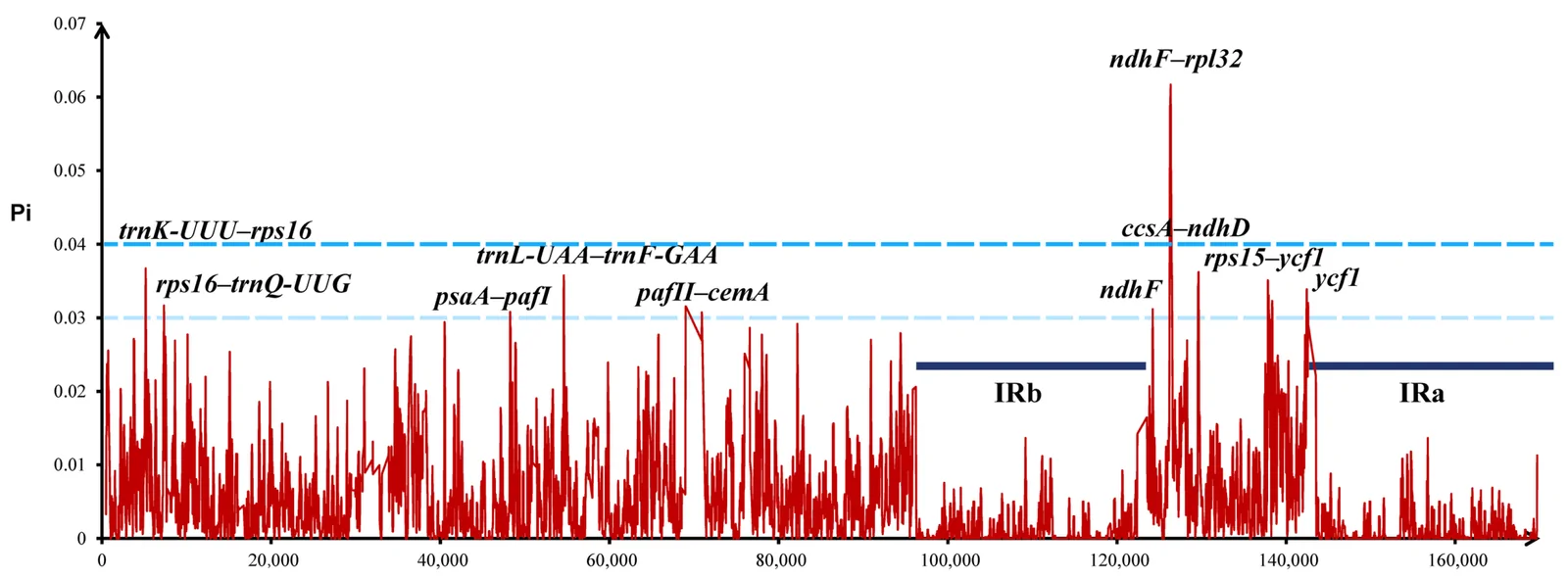
Sliding-window analysis of nucleotide diversity (Pi) across the alignment of 98 *Viburnum* plastomes in Dataset II. The window length was 100 bp, with a step size of 25 bp. The dashed lines indicate Pi values of 0.03 and 0.04, and the five windows exceeding Pi > 0.04 were all located in the *ndhF–rpl32* intergenic region.

Sliding-window analysis was conducted using the aligned complete plastome sequences of 98 *Viburnum* records in Dataset II to evaluate nucleotide diversity across the plastome alignment (Figure 6). Most windows exhibited relatively low nucleotide diversity (Pi), supporting the overall sequence conservation of the sampled *Viburnum* plastomes, although several localized peaks were detected. Using the predefined threshold of Pi > 0.04, five windows were identified, all of which were located within the *ndhF–rpl32* intergenic region in the SSC. The maximum Pi value was 0.06171, and the values of the five windows ranged from 0.04502 to 0.06171 (Table S6). These overlapping windows therefore represented a single major variable region rather than five independent regions. Among the remaining regions, the *trnK-UUU–rps16* and *ccsA–ndhD* intergenic regions exhibited the next highest local peaks, with maximum Pi values of 0.03672 and 0.03624, respectively. Because neither exceeded the predefined threshold, they were not classified as major variable regions. Overall, the results indicate that nucleotide diversity was unevenly distributed across the plastome alignment, with elevated variation concentrated primarily in the *ndhF–rpl32* intergenic region.

### 2.6. Plastome Phylogenetic Relationships

**Figure 7:**
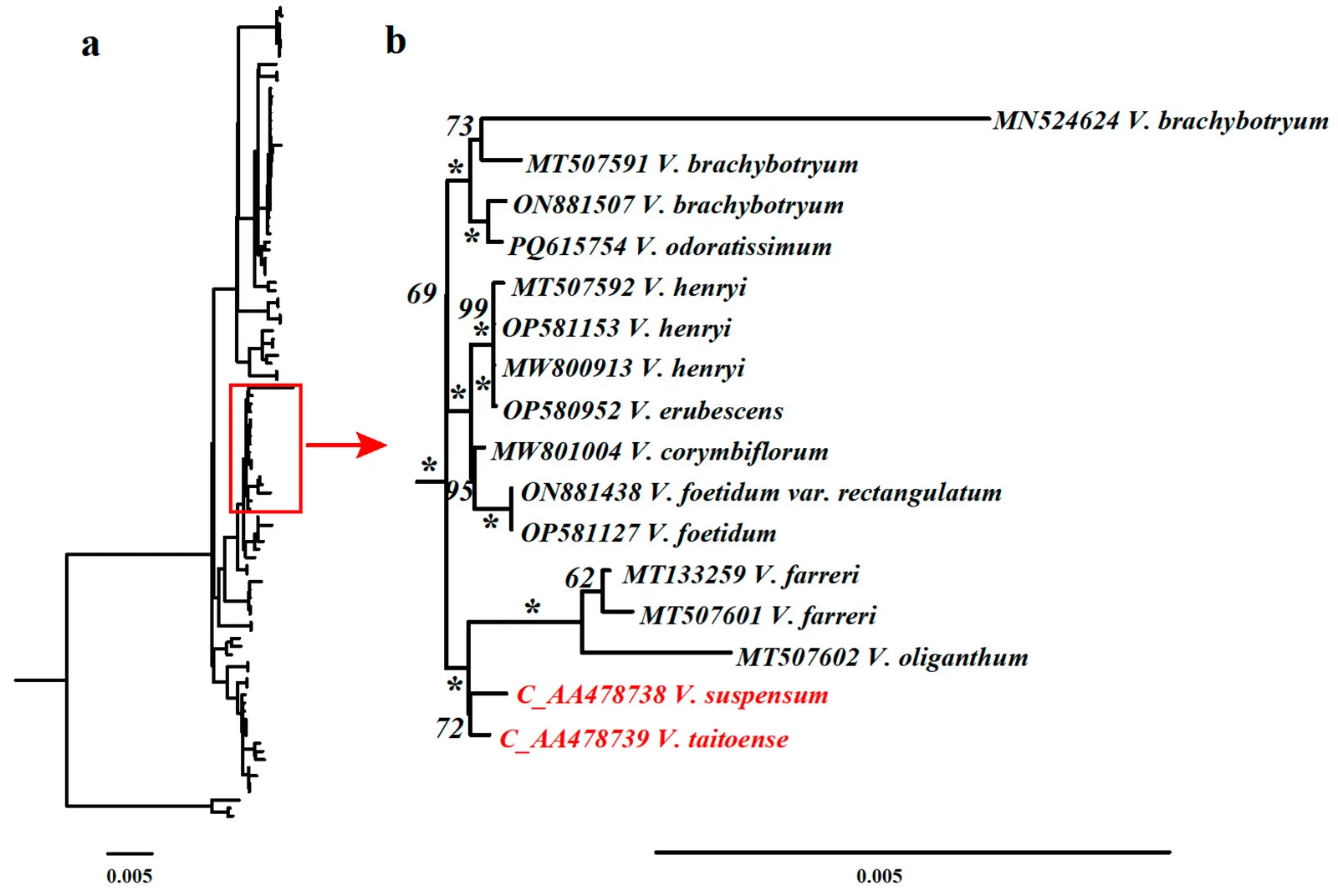
Phylogenetic relationships among *Viburnum* species inferred from complete chloroplast genome sequences. The analysis included 98 *Viburnum* plastome sequences and three *Sambucus* outgroup sequences. (**a**) Maximum likelihood (ML) phylogenetic tree of *Viburnum* inferred from complete chloroplast genome sequences, showing the overall phylogenetic relationships among the sampled taxa. (**b**) Detailed ML phylogenetic relationships of the focal clade containing *V. suspensum* and *V. taitoense*. Bootstrap support values are indicated at the nodes, and an asterisk (*) denotes 100% bootstrap support. The two focal plastomes are highlighted in red.

A maximum likelihood (ML) phylogenetic tree was reconstructed from 101 complete plastome sequences, comprising 98 *Viburnum* plastome sequences and three *Sambucus* outgroup sequences (Figure 7). The resulting tree showed high resolution, with most internal nodes receiving strong bootstrap support, indicating that complete chloroplast genomes provided substantial phylogenetic information for resolving relationships within *Viburnum*.

The sampled plastomes of *Viburnum suspensum* and *V. taitoense* were recovered as a sister pair, although the corresponding node received only moderate bootstrap support (BS = 72). This lineage also included several species traditionally assigned to sect. *Thyrsosma*, including *V. brachybotryum*, *V. odoratissimum*, *V. henryi*, *V. erubescens*, *V. corymbiflorum*, *V. farreri*, and *V. oliganthum*. Notably, *V. foetidum* and *V. foetidum* var. *rectangulatum*, which are traditionally classified into sect. *Odontotinus*, were also nested within this lineage, suggesting partial incongruence between morphology-based sectional classification and chloroplast genome phylogeny.

## 3. Discussion

### 3.1. Plastome Structural Conservation in Viburnum suspensum and V. taitoense

Chloroplast genomes provide valuable molecular resources for evolutionary studies because they combine broadly conserved genomic architectures with lineage-specific variation. Across angiosperms, plastomes commonly retain a quadripartite organization and substantial conservation of gene content and gene order, although structural exceptions occur in multiple lineages [9,23,24]. Comparative analyses within *Viburnum* have revealed a similar pattern across sampled taxa, including conserved genome organization and broadly similar gene complements and gene order [16,17,18]. In the present study, the sampled plastomes of *V. suspensum* and *V. taitoense* exhibited highly similar architectures, including nearly identical genome sizes, the same overall GC content, consistent gene complements, and conserved quadripartite organization. Gene numbers reported for publicly available plastomes should nevertheless be interpreted cautiously because database records may differ in annotation completeness, pipelines, and gene-counting criteria. Consequently, some variation in the gene numbers listed in Table S1 may reflect annotation heterogeneity rather than genuine biological differences among taxa. These values are therefore retained as metadata from the corresponding database records and are not interpreted here as direct evidence of gene-content evolution. The conserved organization of these plastomes is consistent with evolutionary constraints commonly recognized in plastome evolution, but the present comparative data do not identify the mechanisms responsible. The IR regions provide one relevant structural context: early comparative evidence associated loss of the large IR with increased rearrangement, although it did not establish that IR retention universally prevents structural change [25]. Across diverse land plants, genes located in the IR have lower synonymous substitution rates than their single-copy counterparts, supporting an association between IR location and enhanced sequence conservation [26]. Consistent with this broader pattern, the current comparison showed lower sequence divergence in the IR regions than in the LSC and SSC regions. However, IR expansion and contraction can shift junction positions [27], and localized IR-boundary variation has also been documented within *Viburnum* [16,17,18]. Thus, IR-associated conservation should be viewed as compatible with continuing boundary evolution rather than as evidence of structural invariance.

No evident major structural rearrangements were detected in the current comparative analysis, whereas localized differences were observed at the IR/SC junctions and in several sequence regions. The two focal plastomes therefore exhibited broadly conserved genome organization without complete sequence uniformity. In this sense, structural conservation does not imply complete sequence uniformity; rather, it provides the genomic background against which localized sequence divergence can be identified and evaluated. More broadly, comparison of the 54 plastome records in Dataset I extends previous observations of plastome conservation in *Viburnum* while restricting conclusions concerning the two focal species to the plastomes sampled in this study.

### 3.2. Localized Sequence Divergence Within Conserved Viburnum Plastomes

Although plastome architecture is highly conserved, nucleotide variation is not uniformly distributed across the genome. Comparative analyses have shown that substitutions, indels, and phylogenetically informative characters are distributed heterogeneously among plastome regions, even where overall genome organization remains similar [28,29,30]. Published *Viburnum* comparisons reveal the same contrast between a conserved structural framework and localized sequence divergence [16,18]. Thus, the structural conservation established in Section 3.1 provides the genomic context within which localized differences can be recognized.

Intergenic and other noncoding regions often show greater divergence than coding regions and may experience reduced functional constraints, although this distinction is not absolute [28,29]. Consistent with this general pattern, the 54-plastome mVISTA comparison in the present study showed greater divergence in noncoding regions, whereas the IR regions were more conserved than the LSC and SSC regions. In the separate sliding-window analysis of 98 *Viburnum* plastomes, five overlapping windows exceeded the predefined threshold applied here (Pi > 0.04), and all were located in the *ndhF–rpl32* intergenic region, which reached a maximum Pi value of 0.06171. The *trnK-UUU–rps16* and *ccsA–ndhD* intergenic regions showed the next highest local peaks, but their maximum Pi values remained below the predefined threshold. The recovery of *ndhF–rpl32* is consistent with its previously reported variability in comparative angiosperm plastomes [29]. Nevertheless, this region should currently be regarded as a candidate informative region because elevated nucleotide diversity alone does not demonstrate its performance as a molecular marker.

Across published *Viburnum* datasets, localized sequence divergence has repeatedly been detected within an otherwise conserved plastome framework, although the regions identified as most variable differ among studies [16,17,18]. In the present study, the 54-record Dataset I showed broadly conserved plastome organization together with localized sequence differences, whereas sliding-window analysis of the 98-record Dataset II revealed an uneven distribution of nucleotide diversity and identified the *ndhF–rpl32* intergenic region as the only major variable region exceeding the predefined threshold. This heterogeneity does not, by itself, demonstrate mutation-rate acceleration, adaptive evolution, or divergence caused by geographic isolation. Subject to further validation of alignment quality and phylogenetic informativeness, these candidate regions may provide useful sources of plastid variation for future taxonomic and evolutionary studies. Meanwhile, the two focal plastomes exhibited closely similar coding-sequence composition and codon-usage patterns, providing a complementary measure of plastome conservation, as discussed in Section 3.3.

### 3.3. Codon Usage Patterns and Compositional Constraints in Viburnum Plastomes

Although localized sequence divergence occurs within plastomes, coding regions can retain conserved compositional characteristics. *V. suspensum* and *V. taitoense* had nearly identical RSCU profiles, the same set of 29 preferred codons, similar summary ENC values (50.26 and 50.24), and the same reported GC3s (27.7%); 28 preferred codons ended in A or U, whereas UUG was the only preferred codon ending in G. This pattern is consistent with the A/U-ending preference associated with AT-rich plastid coding sequences and reported in other sampled *Viburnum* plastomes [18,31]. The similarity therefore supports conserved codon-composition characteristics between the focal plastomes without implying that their translation systems remained unchanged during diversification.

Considered together, the ENC–GC3s, neutrality, and PR2 analyses indicate that nucleotide composition contributes to codon preference but does not fully explain gene-level variation. The high summary ENC values are consistent with weak overall codon bias, whereas the location of most genes below the ENC–GC3s expectation indicates that GC3s alone is insufficient to account for the observed codon-use concentration [32]. The weak, nonsignificant GC12–GC3 relationships and asymmetric third-position nucleotide usage further argue against a simple composition-only model [33,34]. These combined diagnostics are therefore consistent with contributions from nucleotide composition and additional functional or evolutionary constraints, but they neither determine their relative contributions nor demonstrate that mutation pressure or natural selection predominates.

Codon-use concentration also varied among genes. The consistently low ENC values reported for *psbJ*, *petN*, *rpl36*, *rps8*, and *rpl16* indicate stronger codon-use bias than the plastome-wide average, but do not by themselves establish high expression, translational optimization, or selection. Comparative plastid studies show that gene-specific codon patterns can reflect multiple sequence and evolutionary constraints [31,35,36], providing a plausible but untested context for the focal genes. Together, the similar codon-composition characteristics complement the conserved plastome backbone described in Section 3.1, while the localized sequence divergence identified in Section 3.2 supplies plastid characters that can be evaluated phylogenetically. The resulting phylogeny must nevertheless be interpreted as a history of the plastid genome rather than as a definitive species tree.

### 3.4. Complete Plastomes Provide Phylogenetic Insights into Viburnum Evolution

Early molecular studies established the principal framework of *Viburnum* phylogeny using complementary plastid and nuclear markers. Analyses of the plastid *trnK* intron and nuclear ribosomal ITS recovered several major lineage groups but also identified uncertainty in deeper relationships and in the circumscription of some traditional sections [7]. Independent evidence from the duplicated nuclear gene GBSSI was broadly consistent with parts of this framework, while the root and several supra-sectional relationships remained unresolved [4]. These marker-based studies therefore defined the main phylogenetic context for the genus, but their limited locus sampling constrained resolution of some section boundaries and relationships among closely related taxa.

Plastome-scale datasets subsequently increased the amount of phylogenetic information available from the plastid genome. By combining plastome-scale sequences from representative lineages with broader taxon sampling, Clement et al. improved support for many early branches and established a more explicit phylogenetic classification, although several deep nodes and species complexes remained uncertain [8]. Complete-plastome sampling of Chinese *Viburnum* further clarified relationships among multiple section-level groups [16]. These advances are relevant to the present study because the conserved *Viburnum* plastome backbone still contains localized sequence variation that can contribute phylogenetic signal when analyzed across complete genomes. Nevertheless, a plastome phylogeny represents plastid evolutionary history rather than a definitive species tree. Recent comparisons between plastid and nuclear ribosomal topologies reinforce the importance of evaluating plastome relationships against independent nuclear evidence, without assigning discordance to incomplete lineage sorting, hybridization, introgression, or chloroplast capture in the absence of direct tests [20].

Against this background, our complete-plastome analysis provides a direct plastid comparison between *V. suspensum* and *V. taitoense*. Their plastomes were recovered as a sister pair with moderate bootstrap support (BS = 72), suggesting a close plastid relationship but not establishing a species-level sister relationship. The placement of *V. foetidum* and *V. foetidum* var. *rectangulatum*, traditionally assigned to sect. *Odontotinus*, within a lineage containing taxa assigned to sect. *Thyrsosma* suggests that some morphology-based sectional boundaries warrant further evaluation using independent nuclear evidence. However, the plastome topology alone is insufficient to justify taxonomic revision or determine the evolutionary processes underlying the observed discordance. Overall, localized variation across the sampled *Viburnum* plastomes provides information relevant to the plastid history and phylogenetic placement of the two focal samples.

The present study analyzed one sampled plastome from each focal species. Therefore, the observed similarities and differences characterize these samples but cannot represent the full intraspecific diversity of *V. suspensum* or *V. taitoense*. Broader population-level and geographic sampling, together with nuclear genomic evidence, will be required to evaluate intraspecific variation, geographic differentiation, and species-level relationships.

## 4. Materials and Methods

### 4.1. Plant Materials and Chloroplast Genome Dataset

**Figure 8:**
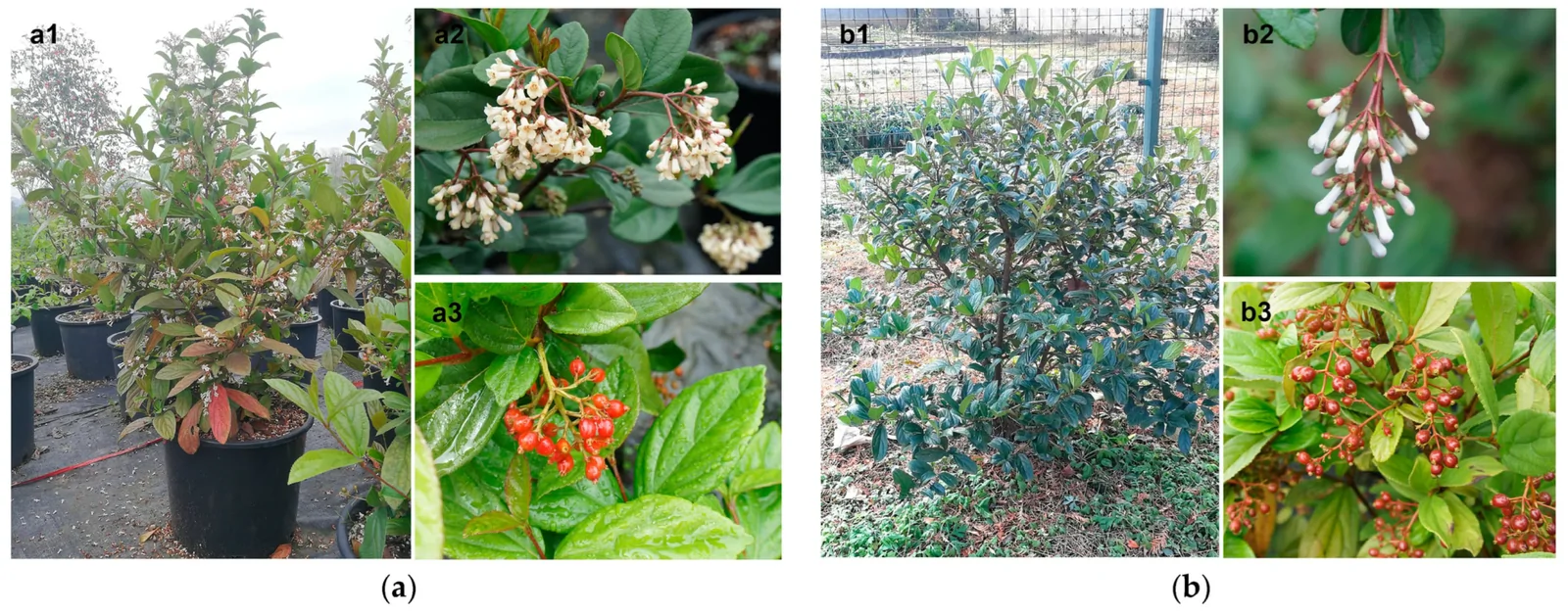
Representative images of *Viburnum suspensum* and *V. taitoense*. (**a**) *V. suspensum*: (**a1**) plant habit, (**a2**) flowering branch, and (**a3**) fruiting branch. (**b**) *V. taitoense*: (**b1**) plant habit, (**b2**) flowering branch, and (**b3**) fruiting branch.

Fresh leaves of *Viburnum suspensum* and *V. taitoense*, originally derived from the Ryukyu Islands and Taiwan, respectively, were obtained from living collections maintained at Hangzhou Botanical Garden, Hangzhou, Zhejiang Province, China (120°07′ E, 30°15′ N) (Figure 8). These materials originated from cultivated accessions of East Asian island lineages and were maintained under ex situ conservation conditions. Voucher specimens were deposited at the Herbarium of Zhejiang Academy of Forestry under voucher numbers VD021 and VD022, respectively. The collected leaf materials were used for chloroplast genome sequencing and subsequent comparative analyses.

In addition to the two newly sequenced chloroplast genomes generated in this study, publicly available plastome sequences of *Viburnum* and related taxa were retrieved from the NCBI GenBank database. Based on different analytical objectives, three datasets with different taxon sampling were constructed.

Three datasets were defined according to the objectives of the respective analyses. Dataset I comprised 54 plastome records, with one representative accession retained for each full taxon label. Infraspecific taxa and the cultivar were treated as distinct entries. This reduced redundancy and prevented taxa represented by multiple accessions from being disproportionately weighted in the broad comparative analyses of plastome structure, sequence conservation, and IR junction variation. Dataset II comprised all 98 *Viburnum* plastome records included in this study. Multiple accessions assigned to the same taxon were retained to capture both intertaxon and within-taxon nucleotide variation in the sliding-window analysis. Dataset III comprised the same 98 *Viburnum* plastome records together with three *Sambucus* plastomes as outgroups, resulting in 101 records for phylogenetic reconstruction.

Detailed information on all plastome sequences, including species names, accession numbers, and dataset assignments, is provided in Supplementary Table S1.

### 4.2. DNA Extraction, Chloroplast Genome Sequencing, Assembly, and Annotation

Total genomic DNA was extracted from silica-gel-dried leaves using a modified cetyltrimethylammonium bromide (CTAB) method. DNA quality and concentration were evaluated by agarose gel electrophoresis (Thermo Fisher Scientific, Waltham, MA, USA) and DNA concentration was measured using a Qubit 2.0 Fluorometer (Thermo Fisher Scientific, Waltham, MA, USA). Approximately 1 μg of high-quality genomic DNA was used for paired-end library construction with an insert size of approximately 350 bp. Paired-end sequencing (PE150) was performed by Novogene Co., Ltd. (Beijing, China) using an Illumina NovaSeq 6000 platform (Illumina, Inc., San Diego, CA, USA), generating approximately 10 Gb of raw data.

The raw sequencing reads were filtered using Trimmomatic v0.39 to remove adapter sequences and low-quality reads [37]. The chloroplast genome was assembled de novo using NOVOPlasty v4.3.1 [38], with the maize (*Zea mays*) chloroplast *rbcL* sequence supplied with the software as the seed sequence (GenBank accession V00171.1). Genome annotation was performed using the CPGAVAS2 online platform [39], followed by manual verification and correction using Geneious Prime 2022.2.1 (Biomatters Ltd., Auckland, New Zealand; https://www.geneious.com, accessed on 1 May 2026) to ensure accurate gene boundaries and annotation. The circular chloroplast genome map was generated using OGDRAW (https://chlorobox.mpimp-golm.mpg.de/OGDraw.html accessed on 1 May 2026). CPGView (http://www.1kmpg.cn/cpgview/ accessed on 1 May 2026) was used for data visualization in this study [40].

### 4.3. Comparative Plastome Analysis

Comparative plastome analyses were conducted using Dataset I. The basic characteristics of each chloroplast genome, including genome size, GC content, gene number, and overall genome organization, were calculated and compared using Geneious Prime 2022.2.1. Whole-genome sequence comparisons among the 54 *Viburnum* plastomes were performed using mVISTA [41], with the complete chloroplast genome of *V. utile* (GenBank accession number: KX792264.1) selected as the reference sequence. The Shuffle-LAGAN mode was applied for sequence alignment, and other parameters were maintained as default settings. The mVISTA output was used to visualize conserved and divergent regions across coding and noncoding sequences. The LSC/IRb (JLB), IRb/SSC (JSB), SSC/IRa (JSA), and IRa/LSC (JLA) junctions of the 54 plastomes in Dataset I were analyzed and visualized using CPJSdraw v1.0.0 [42]. Junction assignments and boundary-associated genes were cross-checked against the corresponding plastome annotations.

For the two newly assembled chloroplast genomes, repeat sequences were identified using Vmatch [43], and the types and distributions of repeats were compared among the analyzed plastomes. Repeat sequences were searched across the complete plastome, including both IRa and IRb. Only the single long inverted-repeat pair corresponding to the canonical IRa–IRb structure was excluded. Shorter repeat pairs located within the IR regions, including mirror-associated records arising from the duplicated IR copies, were retained and counted as separate software-detected repeat pairs. Chloroplast simple sequence repeats (cpSSRs) were identified using the MISA (MIcroSAtellite identification tool) software [44] with the following thresholds: mononucleotide repeats ≥10, dinucleotide repeats ≥5, and tri- to hexanucleotide repeats ≥4. The maximum interruption distance for compound SSRs was set to 100 bp.

### 4.4. Nucleotide Diversity and Sliding-Window Analysis

The complete plastome sequences in Dataset II were aligned using MAFFT v7.463 [45], and the alignment was manually inspected and refined in BioEdit v7.2.5 [46]. Singleton gap or InDel patterns occurring in only one sequence were treated as potential alignment artefacts and excluded during manual refinement, whereas InDel patterns supported by at least two sequences were retained in the curated alignment. Ambiguously aligned regions that could not be reliably resolved by manual inspection were removed before nucleotide-diversity analysis. Nucleotide diversity (Pi) was subsequently calculated in DnaSP v6 [47], with alignment sites containing gaps or missing data excluded from the Pi calculation. Sliding-window analysis was performed using a window length of 100 bp and a step size of 25 bp. Windows with Pi > 0.04 were identified as major variable windows. All window coordinates reported in Figure 6 and Table S6 refer to positions in the multiple-sequence alignment and should not be interpreted as physical coordinates in an individual plastome.

### 4.5. Codon Usage Bias Analysis

Codon usage patterns were evaluated using the protein-coding sequences (CDSs) extracted from the two newly assembled chloroplast genomes. After removing duplicated coding sequences, 81 unique protein-coding genes were retained for codon-usage analysis. Because no valid ENC estimate was obtained for *psbT*, the ENC–GC3s plot and the quantitative comparison with the expected ENC curve were based on the remaining 80 genes in each plastome. Codon usage parameters, including the effective number of codons (ENC), relative synonymous codon usage (RSCU), and GC content at the third position of synonymous codons (GC3s), were calculated using CodonW v1.4.2 (http://codonw.sourceforge.net accessed on 01 August 2026). The nucleotide composition of each protein-coding gene was further analyzed using BioDataTools (https://www.biodatatools.cn accessed on 01 August 2026), including the GC contents at the first, second, and all third codon positions (GC1, GC2, and GC3) and the overall GC content of coding sequences (GCall). The total codon counts, including stop codons, were 26,971 and 26,965 for *V. suspensum* and *V. taitoense*, respectively. Stop codons were excluded from the identification and enumeration of preferred codons based on RSCU values.

To investigate the factors influencing codon usage bias, ENC-GC3s, neutrality, and parity rule 2 (PR2) analyses were performed. The ENC-GC3s plot was generated by plotting ENC values against GC3s values, with the expected curve representing codon usage bias driven solely by nucleotide composition [32,48]. Neutrality analysis was conducted by examining the relationship between GC12 and GC3, where GC3 was plotted against GC12 to evaluate the relative contributions of mutational pressure and selective constraints on codon usage patterns [33]. PR2 bias analysis was performed by plotting G3/(G3 + C3) against A3/(A3 + U3) to assess the influence of mutation and selection on nucleotide distribution at synonymous third codon positions [49]. All statistical analyses and graphical representations were performed using R v4.3.2. The expected ENC value was calculated according to the Wright equation: ENC_expected = 2 + GC3s + 29/[GC3s^2^ + (1 − GC3s)^2^].

### 4.6. Phylogenetic Analysis of Complete Plastomes

Phylogenetic reconstruction was performed using Dataset III, which comprised 101 complete plastome sequences: 98 *Viburnum* plastome sequences and three *Sambucus* outgroup sequences. The plastome sequences were aligned using MAFFT v7.463 [45] with the automatic strategy selection option, and ambiguously aligned regions were removed using trimAl v1.2 [50]. Because the complete plastome was analyzed as a single linked organellar dataset to evaluate its overall superbarcode signal, the alignment was not partitioned by gene or codon position. Instead, the best-fitting substitution model was selected for the complete alignment. The optimal nucleotide substitution model was determined using ModelFinder based on the Bayesian Information Criterion (BIC) [51], and the GTR + F + I model was selected as the best-fit model. Subsequently, the maximum-likelihood (ML) phylogenetic tree was inferred using IQ-TREE v1.6.8 [52] under the selected GTR + F + I model. Branch support was evaluated using 5000 bootstrap replicates. The resulting phylogenetic tree was visualized and edited using FigTree v1.4.4 (https://tree.bio.ed.ac.uk/software/figtree.com accessed on 1 August 2026).

## 5. Conclusions

The sampled plastomes of *V. suspensum* and *V. taitoense* exhibited conserved genome organization together with localized sequence divergence. They were 158,577 bp and 158,544 bp in length, respectively, and each contained 133 annotated gene features with the same overall GC content of 38.1%. Comparisons across the 54 plastome records in Dataset I revealed broadly conserved genome organization and variation in IR-boundary configurations, whereas sliding-window analysis of the 98-record Dataset II identified the *ndhF–rpl32* intergenic region as the only major variable region exceeding the predefined threshold (maximum Pi = 0.06171). The two focal plastomes also exhibited similar codon-usage characteristics, including 29 preferred codons and weak overall codon bias (ENC = 50.26 and 50.24). Plastome phylogenetic analysis based on Dataset III recovered the two focal plastomes as a sister pair with moderate bootstrap support (BS = 72), providing tentative evidence for a close plastid relationship without establishing a species-level sister relationship. Collectively, these results characterize plastome conservation, localized sequence divergence, codon usage, and plastome-based phylogenetic placement within the defined sampling framework. Broader population-level sampling and nuclear genomic evidence will be required to evaluate intraspecific diversity, geographic differentiation, and species-level relationships.

## Figures and Tables

**Table 1:** Functional classification of genes in the chloroplast genome of *Viburnum* species.

Category of Genes	Group of Genes	Name of Genes	Unique Genes
**Self-replication**	Large subunit of ribosome	*rpl2* ᵃ(×2), *rpl14*, *rpl16* ᵃ, *rpl20*, *rpl22*, *rpl23*(×2), *rpl32*, *rpl33*, *rpl36*	9
	Small subunit of ribosome	*rps2*, *rps3*, *rps4*, *rps7*(×2), *rps8*, *rps11*, *rps12* ᶜ, *rps14*, *rps15*, *rps16* ᵃ, *rps18*, *rps19*	12
	DNA-dependent RNA polymerase	*rpoA*, *rpoB*, *rpoC1*ᵃ, *rpoC2*	4
	rRNA genes	*rrn4.5*(×2), *rrn5*(×2), *rrn16*(×2), *rrn23*(×2)	4
	tRNA genes	*trnA*-UGC ᵃ(×2), *trnC*-GCA, *trnD*-GUC, *trnE*-UUC, *trnF*-GAA, *trnG*-GCC, *trnG*-UCC ᵃ, *trnH*-GUG, *trnI*-GAU ᵃ(×2), *trnK*-UUU ᵃ, *trnL*-CAA(×2), *trnL*-UAA ᵃ, *trnL*-UAG, *trnfM*-CAU, *trnM*-CAU(×3), *trnN*-GUU(×2), *trnP*-UGG, *trnQ*-UUG, *trnR*-ACG(×2), *trnR*-UCU, *trnS*-GCU, *trnS*-GGA, *trnS*-UGA, *trnT*-GGU, *trnT*-UGU, *trnV*-GAC(×2), *trnV*-UACᵃ, *trnW*-CCA, *trnY*-GUA	29
**Photosynthesis**	Photosystem I	*psaA*, *psaB*, *psaC*, *psaI*, *psaJ*, *psaM*	6
	Photosystem II	*psbA*, *psbB*, *psbC*, *psbD*, *psbE*, *psbF*, *psbH*, *psbI*, *psbJ*, *psbK*, *psbL*, *psbM*, *psbT*, *psbZ*	14
	Photosystem assembly factors	*pafI* ^b^, *pafII*, *pbf1*	3
	NADH dehydrogenase	*ndhA* ᵃ, *ndhB* ᵃ(×2), *ndhC*, *ndhD*, *ndhE*, *ndhF*, *ndhG*, *ndhH*, *ndhI*, *ndhJ*, *ndhK*	11
	Cytochrome b/f complex	*petA*, *petB* ᵃ, *petD* ᵃ, *petG*, *petL*, *petN*	6
	ATP synthase	*atpA*, *atpB*, *atpE*, *atpF* ᵃ, *atpH*, *atpI*	6
	Large subunit of Rubisco	*rbcL*	1
**Other genes**	Protease	*clpP* ^b^	1
	Maturase	*matK*	1
	Translation initiation factor	*infA*	1
	Envelope membrane protein	*cemA*	1
	Subunit of acetyl-CoA carboxylase	*accD*	1
	c-type cytochrome synthesis gene	*ccsA*	1
	Conserved open reading frames	*ycf1*(×2), *ycf2*(×2), *ycf15*(×2)	3
**Total number of unique genes**			114

^a^ indicates genes containing a single intron; ^b^ indicates genes containing two introns; ^c^ indicates the trans-spliced *rps12* gene, which comprises one 5′ exon in the LSC region and a duplicated 3′ exon region in the two IRs, with each 3′ region containing exons 2 and 3. The duplicated 3′ regions do not represent two complete copies of *rps12*.

**Table 2:** Distribution of repeat pairs and functional locations of repeat occurrences in the chloroplast genomes of *V. suspensum* and *V. taitoense*.

Category	Subcategory	*V. suspensum*	*V. taitoense*
**Total repeat pairs**	—	45	44
**Repeat type (repeat pairs)**	Direct repeats	19	18
	Palindromic repeats	26	26
**Genomic distribution (repeat pairs)**	LSC	12	11
	SSC	1	1
	IR	22	22
	Cross-region	10	10
**Functional location (repeat occurrences)**	CDS	38	38
	Intron	20	20
	tRNA	2	2
	rRNA	0	0
	Intergenic region	26	24
	Feature boundary	4	4
**Total repeat occurrences**	—	90	88

Each repeat pair comprises two occurrences. Repeat type and genomic distribution were summarized at the repeat-pair level (45 and 44 pairs), whereas functional locations were summarized separately for all repeat occurrences (90 and 88 occurrences). Repeat pairs with both occurrences located in the same major plastome region were assigned to that region; pairs with occurrences located in different regions, or with an occurrence spanning a region boundary, were classified as cross-region. IRa and IRb were treated collectively as the IR category for this regional summary. A repeat occurrence crossing two functional regions was classified as a feature-boundary occurrence.

**Table 3:** Distribution and composition of simple sequence repeats (SSRs) in the chloroplast genomes of *V. suspensum* and *V. taitoense*.

Category	Subcategory	*V. suspensum*	*V. taitoense*
**Total SSRs**		42	41
**Motif composition**	A/T	41	40
	C/G	1	1
**Genomic region**	LSC	30	27
	SSC	6	8
	IR	6	6
**Functional location**	CDS	11	12
	Intron	4	5
	tRNA	0	1
	rRNA	0	0
	Intergenic region	27	23

All identified SSRs were mononucleotide repeats.

## Data Availability

The complete chloroplast genome sequences of *V. suspensum* and *V. taitoense* generated in this study have been deposited in the GenBase database of the China National Center for Bioinformation (CNCB-NGDC) under accession numbers C_AA478738.1 and C_AA478739.1, respectively, and are accessible via the link: https://ngdc.cncb.ac.cn/genbase/review/c99ce05416df(accessed on 19 August 2026).

## Supplementary Materials

The following supporting information can be downloaded at https://www.mdpi.com/article/10.3390/ijms27219467/s1.
