# Supplementary material for: Comparative Plastome Analysis of Two Viburnum Species from East Asian Islands: Genome Conservation, Localized Divergence, and Plastome-Based Phylogenetic Placement
Source: Int J Mol Sci. 2026 Oct 23;27(21):9467. doi: 10.3390/ijms27219467 (PMC13763542; doi:10.3390/ijms27219467)
Supplement: Supplementary file 1 [file ijms-27-09467-s001.zip › ijms-4573207-supplementary.pdf]

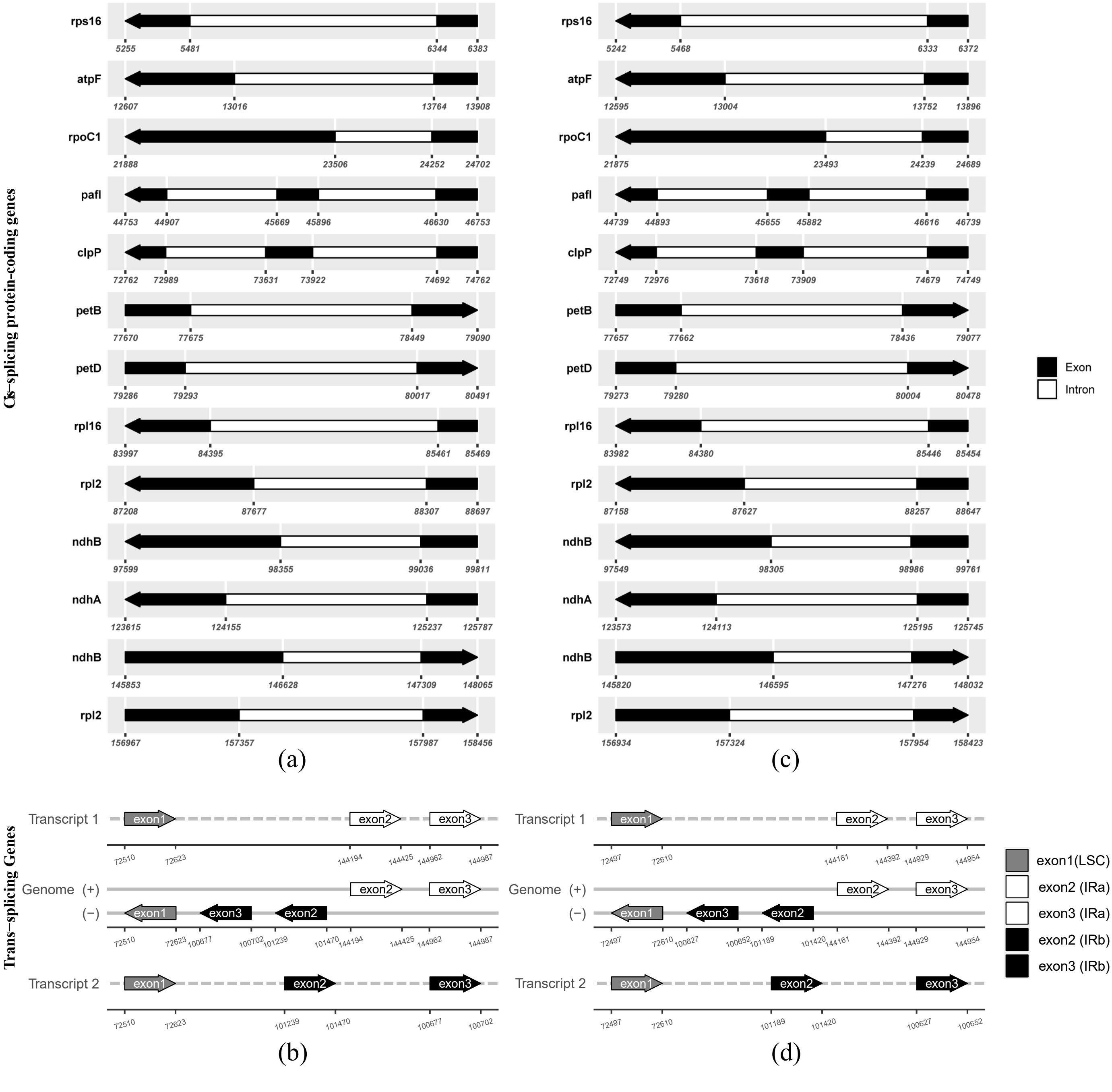
Cis−splicing protein-coding genes
rps16
5255 5481 6344 6383
atpF
12607 13016 13764 13908
rpoC1
21888 23506 24252 24702
pafI
44753 44907 45669 45896 46630 46753
clpP
72762 72989 73631 73922 74692 74762
petB
77670 77675 78449 79090
petD
79286 79293 80017 80491
rpl16
83997 84395 85461 85469
rpl2
87208 87677 88307 88697
ndhB
97599 98355 99036 99811
ndhA
123615 124155 125237 125787
ndhB
145853 146628 147309 148065
rpl2
156967 157357 157987 158456
(a)
rps16
5242 5468 6333 6372
atpF
12595 13004 13752 13896
rpoC1
21875 23493 24239 24689
pafI
44739 44893 45655 45882 46616 46739
clpP
72749 72976 73618 73909 74679 74749
petB
77657 77662 78436 79077
petD
79273 79280 80004 80478
rpl16
83982 84380 85446 85454
rpl2
87158 87627 88257 88647
ndhB
97549 98305 98986 99761
ndhA
123573 124113 125195 125745
ndhB
145820 146595 147276 148032
rpl2
156934 157324 157954 158423
(c)
Exon
Intron
Trans−splicing Genes
Transcript 1
exon1 exon2 exon3
72510 72623 144194 144425 144962 144987
Genome (+)
exon2 exon3
(−)
exon1 exon3 exon2
72510 72623 100677 100702 101239 101470 144194 144425 144962 144987
Transcript 2
exon1 exon2 exon3
72510 72623 101239 101470 100677 100702
(b)
Transcript 1
exon1 exon2 exon3
72497 72610 144161 144392 144929 144954
Genome (+)
exon2 exon3
(−)
exon1 exon3 exon2
72497 72610 100627 100652 101189 101420 144161 144392 144929 144954
Transcript 2
exon1 exon2 exon3
72497 72610 101189 101420 100627 100652
(d)
exon1(LSC)
exon2 (IRa)
exon3 (IRa)
exon2 (IRb)
exon3 (IRb)

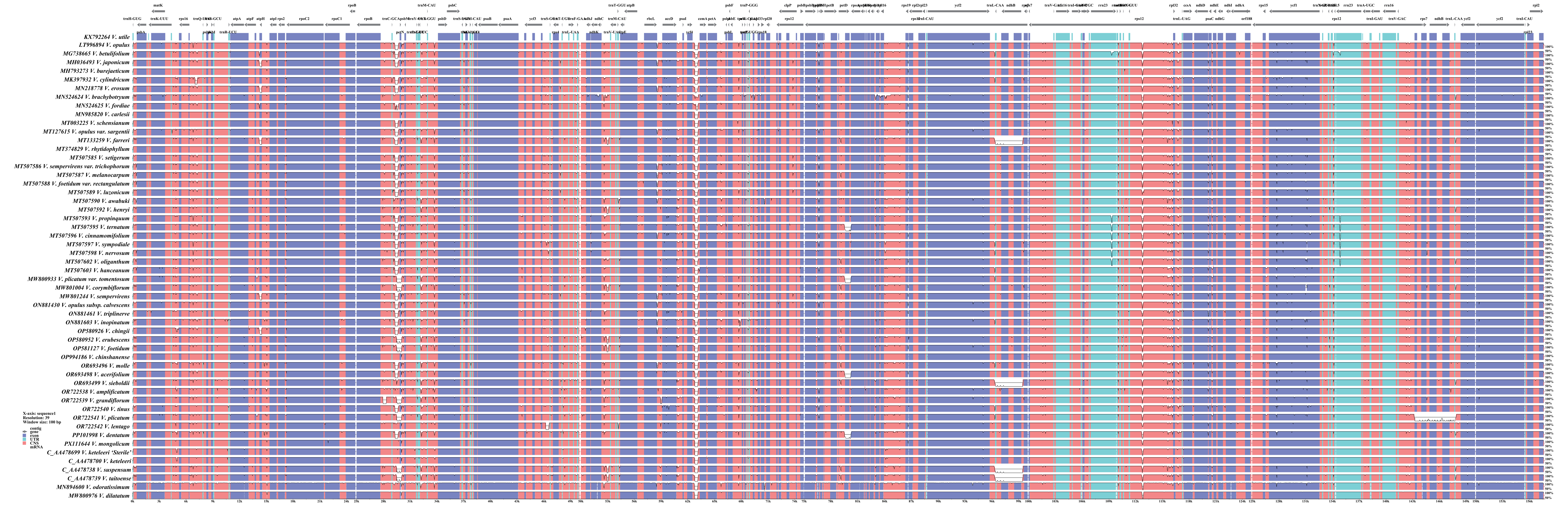
KX792264 V. utile
LT996894 V. opulus
MG738665 V. betulifolium
MH036493 V. japonicum
MH793273 V. burejaeticum
MK397932 V. cylindricum
MN218778 V. erosum
MN524624 V. brachybotryum
MN524625 V. fordiae
MN985820 V. carlesii
MT003225 V. schensianum
MT127615 V. opulus var. sargentii
MT133259 V. farreri
MT374829 V. rhytidophyllum
MT507585 V. setigerum
MT507586 V. sempervirens var. trichophorum
MT507587 V. melanocarpum
MT507588 V. foetidum var. rectangulatum
MT507589 V. luzonicum
MT507590 V. awabuki
MT507592 V. henryi
MT507593 V. propinquum
MT507595 V. ternatum
MT507596 V. cinnamomifolium
MT507597 V. sympodiale
MT507598 V. nervosum
MT507602 V. oliganthum
MT507603 V. hanceanum
MW800933 V. plicatum var. tomentosum
MW801004 V. corymbiflorum
MW801244 V. sempervirens
ON881430 V. opulus subsp. calvescens
ON881461 V. triplinerve
ON881603 V. inopinatum
OP580926 V. chingii
OP580952 V. erubescens
OP581127 V. foetidum
OP994186 V. chinshanense
OR693496 V. molle
OR693498 V. acerifolium
OR693499 V. sieboldii
OR722538 V. amplificatum
OR722539 V. grandiflorum
OR722540 V. tinus
OR722541 V. plicatum
OR722542 V. lentago
PP101998 V. dentatum
PX111644 V. mongolicum
C_AA478699 V. keteleeri 'Sterile'
C_AA478700 V. keteleeri
C_AA478738 V. suspensum
C_AA478739 V. taitoense
MN894600 V. odoratissimum
MW800976 V. dilatatum
X-axis: sequence1
Resolution: 39
Window size: 100 bp
contig
gene
exon
UTR
CNS
mRNA

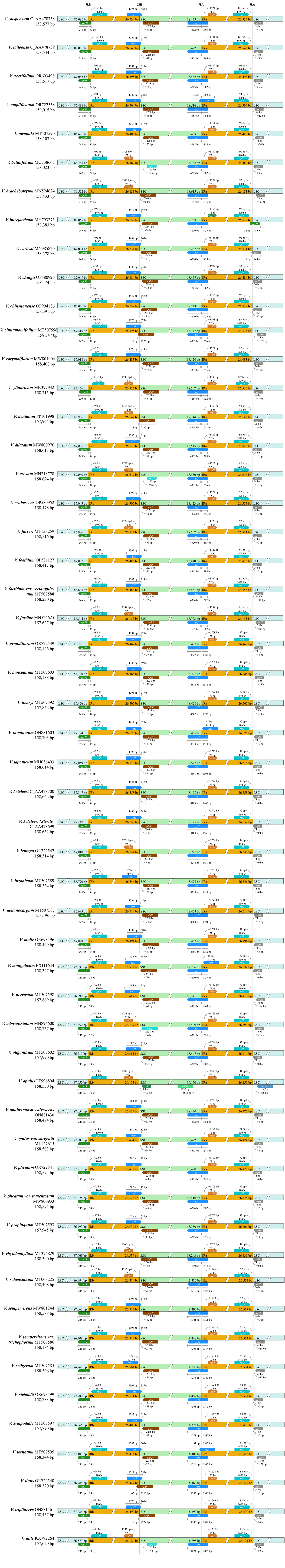

JLB
JSB
JSA
JLA

Supplementary Figures Legend

Figure S1. Exon–intron structures of intron-containing protein-coding genes in the chloroplast genomes of *V. suspensum* and *V. taitoense*. (a) Cis-splicing protein-coding genes of *V. suspensum*. (b) Trans-splicing structure of *rps12* in *V. suspensum*. (c) Cis-splicing protein-coding genes of *V. taitoense*. (d) Trans-splicing structure of *rps12* in *V. taitoense*. The six intron-containing tRNA genes listed in Table 1 are not shown.

Figure S2. Comparative analysis of 54 *Viburnum* plastomes using mVISTA, with *V. utile* (KX792264.1) as the reference.

Figure S3. Comparison of the LSC/IRb, IRb/SSC, SSC/IRa, and IRa/LSC junctions among 54 *Viburnum* plastomes analyzed and visualized using CPJSdraw v1.0.0.
